# Successful bone marrow transplantation in a patient with DNA ligase IV deficiency and bone marrow failure

**DOI:** 10.1186/1750-1172-2-5

**Published:** 2007-01-15

**Authors:** Bernd Gruhn, Joerg Seidel, Felix Zintl, Raymonda Varon, Holger Tönnies, Heidemarie Neitzel, Astrid Bechtold, Holger Hoehn, Detlev Schindler

**Affiliations:** 1 Department of Pediatrics, University of Jena, Kochstr. 2, D-07740 Jena, Germany; 2 Department of Human Genetics, Charité – Campus Virchow-Klinikum, Augustenburger Platz 1, Humboldt University Berlin, 13353 Berlin, Germany; 3 Department of Human Genetics, University of Wurzburg, Biozentrum, Am Hubland, 97074 Wurzburg, Germany; 4 Department of Human Genetics, University of Jena, D-07740 Jena, Germany

## Abstract

**Background:**

DNA Ligase IV deficiency syndrome is a rare autosomal recessive disorder caused by hypomorphic mutations in the DNA ligase IV gene (*LIG4*). The clinical phenotype shows overlap with a number of other rare syndromes, including Seckel syndrome, Nijmegen breakage syndrome, and Fanconi anemia. Thus the clinical diagnosis is often delayed and established by exclusion.

**Methods:**

We describe a patient with pre- and postnatal growth retardation and dysmorphic facial features in whom the diagnoses of Seckel-, Dubowitz-, and Nijmegen breakage syndrome were variably considered. Cellular radiosensitivity in the absence of clinical manifestations of Ataxia telangiectasia lead to the diagnosis of DNA ligase IV (LIG4) deficiency syndrome, confirmed by compound heterozygous mutations in the *LIG4 *gene. At age 11, after a six year history of progressive bone marrow failure and increasing transfusion dependency the patient was treated with matched sibling donor hematopoetic stem cell transplantation (HSCT) using a fludarabine-based conditioning regimen without irradiation.

**Results:**

The post-transplantation course was uneventful with rapid engraftment leading to complete and stable chimerism. Now at age 16, the patient has gained weight and is in good clinical condition.

**Conclusion:**

HSCT using mild conditioning without irradiation qualifies as treatment of choice in LIG4-deficient patients who have a matched sibling donor.

## Background

DNA Ligase IV deficiency syndrome is a rare autosomal recessive disorder caused by hypomorphic mutations in the DNA ligase IV gene (*LIG4*) [1]. The gene product of the *LIG4 *gene functions in nonhomologous end-joining (NHEJ), a major repair pathway for DNA double-strandbreaks in mammalian cells that is activated following DNA damage, but also during class switch [2] and during V(DJ) recombination [3]. The clinical phenotype shows overlap with a number of other rare syndromes, including Seckel syndrome, Nijmegen breakage syndrome, and Fanconi anemia. As a result, the clinical diagnosis is often delayed and established by exclusion. LIG4-deficient patients are characterized by microcephaly, growth retardation starting *in utero*, distinctive facial appearance ("bird-like face"), developmental delay, immunodeficiency, pancytopenia, and pronounced clinical and cellular radiosensitivity [1,4,5]. According to their radiosensitive cellular phenotype, LIG4-deficient patients belong to the group of human radiosensitivity syndromes which include ataxia telangiectasia (AT), Nijmegen breakage syndrome (NBS), Rad 50 and Mre 11 deficiency, thrombocytopenia absent radii syndrome (TAR), Artemis syndrome, and Cernunnos-XLF syndrome [1,5-8]. LIG4-deficient patients share features with the genetic instability syndrome Fanconi anemia (FA), including growth failure, bone marrow failure and increased risk of leukemia [5]. FA patients are successfully treated by hematopoietic stem cell transplantation (HSCT), preferably from matched sibling donors [9-13], whereas HSCT has only rarely been applied in patients with radiosensitivity syndromes [11]. We present the clinical course of a LIG4-deficient patient who is in good condition five years after a matched sibling donor bone marrow transplantation (BMT).

## Case report

**Figure 1:**
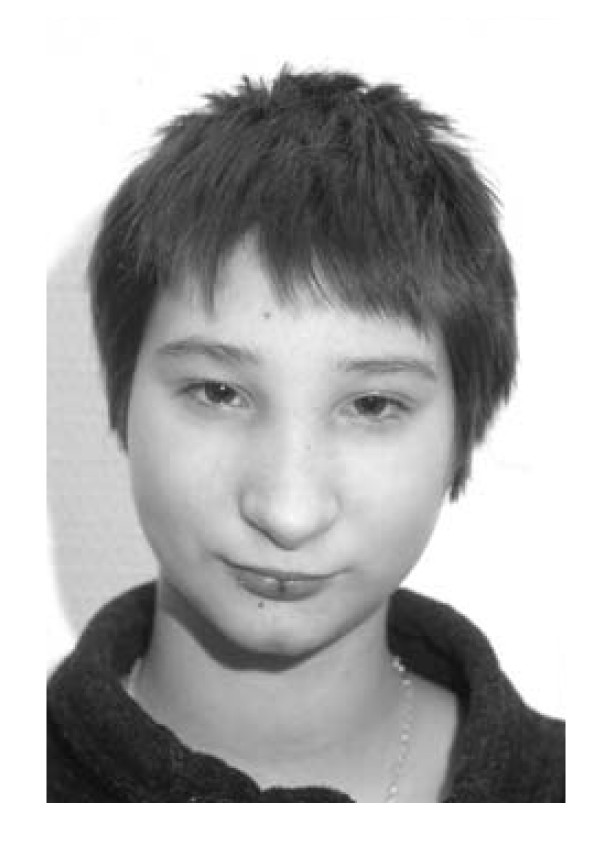
Patient at age 12 showing dsymorphic features including microcephaly (head circumference 43 cm), hypertelorism, broad nasal bridge, broadened tip of the nose, and thin upper lip. A photograph of this patient and the description of her mutations have previously been presented in figure 2 of the report by O'Driscoll *et al*. [4]. It should be noted that there is a labeling error in the legend of figure 2 in reference [4]: The text corresponding to the photograph of our patient is marked with (B) but should read (C).

**Figure 2:**
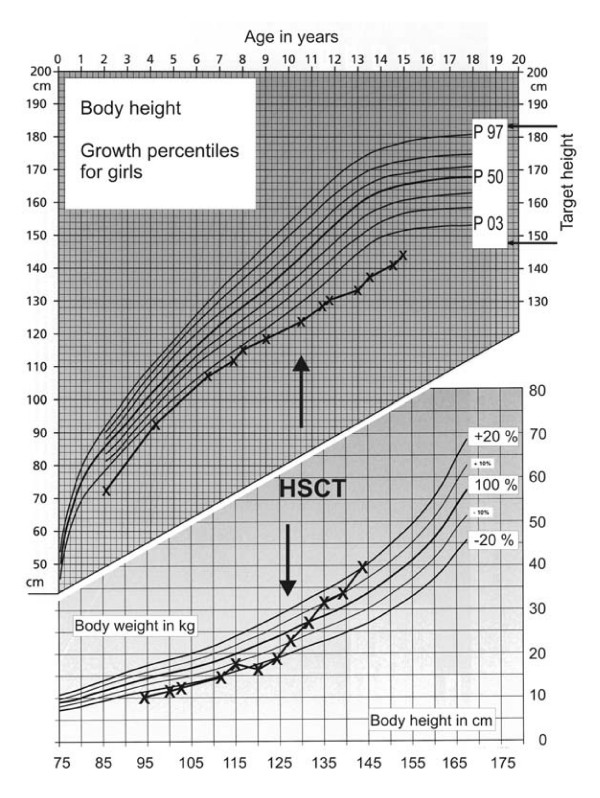
Height and weight prior to and after hematopoietic stem cell transplantation (HSCT).

**Figure 3:**
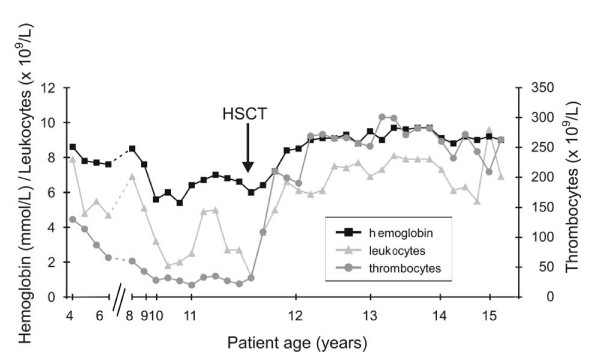
Time course of hematological parameters prior to and after hematopoietic stem cell transplantation (HSCT). Broken lines denote lack of data.

**Figure 4:**
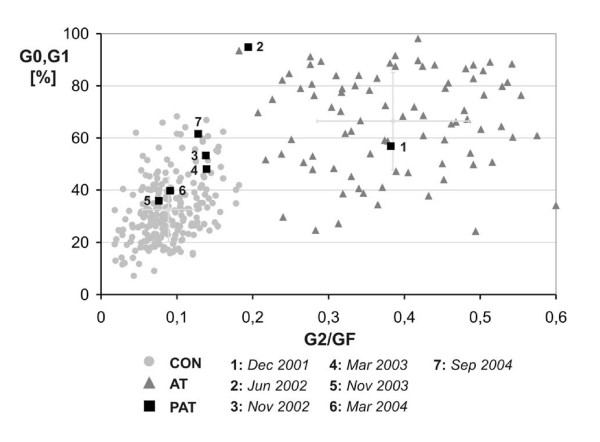
Determination of mitogen response (G0/G1) and radiosensitivity (G2/GF) via bivariate flow cytometry. Circles: radiation resistant cases (controls); Triangles: radiosensitive cases (ataxia telangiectasia, AT). Squares: patient blood samples analyzed at different time points prior to (**1**) and after HSCT (**2**–**7**). BMT was performed in May 2002. Immunosuppression following BMT caused lack of mitogen response (time point **2**).

The patient is the second of three children of healthy, non-consanguineous parents. There was no history of hereditary disorders in the family. During the 22^nd ^week of pregnancy sonography suggested microcephaly and severe growth retardation. Spontaneous uncomplicated delivery occured at the 35^th ^week. The baby was small for gestational age (42 cm), and birth weight was 1500 g. Head circumference was not recorded but described as severely reduced. Developmental milestones were somewhat delayed: The girl started walking at the age of 15 months and began to use single words at the age of 18 months. Because of her pronounced microcephaly, short stature, psychomotoric delay and her distinctive facial appearance ("bird-like face"; cf. figure 1) she received the tentative diagnosis of Seckel syndrome. Her growth velocity remained below the third percentile (figure 2). Starting at age five the child developed thrombocytopenia and anemia followed by leukocytopenia (figure 3). A combination of pancytopenia with features like pre- and postnatal growth retardation, telecanthus, epicanthal folds, ptosis, and broadening of the bridge and tip of the nose similar to our patient had been previously described in two children with Dubowitz syndrome [14,15] leading to consideration of this diagnosis. Our patient increasingly suffered from recurrent infections of the inner ear and respiratory tract with low immunoglobulin levels, requiring frequent hospital admissions. Bone marrow histology (at age 8) showed hypoplastic marrow with cellular dysplasia. This finding was remarkable since cytopenia in LIG4-deficient patients has previously been attributed to autoimmunity rather than to a primary bone marrow defect [16]. Standard chromosome analysis revealed a female karyotype (46, XX) without evidence for numerical aberrations. There were no signs of monosomy 7 or trisomy 8 in bone marrow preparations, but chromosome breakage studies of peripheral blood mononuclear cells showed increased spontaneous breakage. Bleomycin-treatments of 72 h peripheral blood mononuclear cell cultures yielded strongly increased breakage rates (cf. table 1), suggesting a radiosensitive cellular phenotype and the diagnosis of Nijmegen breakage syndrome. However, no mutations were found in the nibrin gene. In order to confirm the cellular radiosensivity phenotype, cell cycle analysis using bivariate BrdU-Hoechst ethidium bromide flow cytometry was performed. As illustrated in figure 4, following irradiation with 1.5 Gy and 72 h *in vitro *culture, the patients' mononuclear blood cells proved as radiosensitive as cells from patients with ataxia telangiectasia. Given a normal serum alpha-fetoprotein (AFP) level and the absence of clinical features of AT, the diagnosis of AT was considered unlikely, but diagnostic considerations were extended to the AT-like syndromes which include the DNA ligase IV deficiency syndrome. As reported previously [1] mutation analysis of the *LIG4 *gene revealed the mutations G469E (maternal) and R814X (paternal), confirming the diagnosis of DNA ligase IV deficiency syndrome.

**Table 1:** 

Bleomycin **μ**g/ml	0	0.1	0.25	0.5
negative control	0.06	0.48	0.5	0.98
patient	0.02	1.04	1.28	1.78
positive control	0.06	1.33	1.52	2.4

Chromosome breakage rates (percent breaks per cell) of peripheral blood mononuclear cells exposed to the respective concentrations of the radiomimetic bleomycin for four hours. Negative control = blood samples from healthy donors; positive control = blood sample from a proven AT patient.

### Hematopoietic stem cell transplantation

As illustrated in figure 3, between ages 10 and 11 years the patient's peripheral blood counts showed continuous decline requiring substitution with several erythrocyte concentrates. Bone marrow aspiration revealed strongly reduced cellularity with very few megakaryocytes confirming progression of bone marrow hypoplasia such that there was concern of beginning bone marrow failure (8% myelocytes, 9% band granulocytes, 9% segmented granulocytes, 2% monocytes, 50% erythroblasts, 20% lymphocytes). Due to poor cellularity a repeat bone marrow chromosome study was not attempted. Because of the patient's continuously decreasing peripheral blood counts, deteriorating general condition, and the known risk of LIG4-deficient patients for hematologic malignancies [5,17], the option of a timely BMT was considered. Her 15-year-old HLA-identical brother was healthy and carried no ligase IV mutation, making him available as donor and facilitating the decision in favor of transplantation. On admission to transplant, the patient's white blood cell (WBC) count was 2.7 × 10^9^/L, platelets were 22 × 10^9^/L, and hemoglobin was 6.6 mmol/L. Immunoglobulin (Ig) levels before transplantation were IgM 0.35 (g/L), IgG 8.83 (g/L), and IgA 0.74 (g/L). Lymphocyte subsets were CD3 960, CD4 412, CD8 531, CD19 11, and NK 222 (cells/microlitre). Total lymphocytes (CD45+) were 98.1%, monocytes (CD14/45+) were 1.1%, NK cells (CD16+56+) were 17.8%, and T cells (CD3+) were 76.8%.

Taking into account the increased cellular radiosensitivity of our patient, conditioning was ablative yet mild, closely following the protocol of McCloy *et al*. [18]. Conditioning consisted of fludarabine (30 mg/m^2^/day for 4 days), cyclophosphamide (10 mg/kg/day for 4 days), and antithymocyte globulin, ATG (15 mg/kg for 4 days). Although ATG is not essential in transplants with a fully matched sibling donor it was given in order to secure both engraftment and graft versus host disease (GvHD) prophylaxis in the absence of metothrexate. In order to avoid possible complications of radiosensitivity, the conditioning regimen did not include irradiation [18].

The patient received 5.43 × 10^8^/kg nucleated cells of unmanipulated bone marrow from her HLA-identical brother. GvHD prophylaxis consisted of cyclosporine A at 3 mg/kg/d from day -1 until day 64 but without additional methotrexate. On day 5, granulocyte colony- stimulating factor (G-CSF) (5 **μ**g/kg/day) was started and given until day 22. The patient did not suffer from any severe side effects and did not even loose her hair. The leukocyte nadir was reached on day -1 with 0.1 × 10^9^/L. Engraftment was observed as early as day 10 with an absolute neutrophile count (ANC) of > 0.5 × 10^9^/L (74% segmented granulocytes; 10% band granulocytes) and a WBC of 1.1 × 10^9^/L. Prophylaxis for fungal, viral and bacterial infections was with acyclovir (30 mg/kg/day from day -2 until day 11), liposomal amphotericin B (1 mg/kg/day from day 6 until day 10), fluconazol (5 mg/kg/day from day 12 until day 16), metronidazol (20 mg/kg/day until day 11), ceftacidim (100 mg/kg/day until day 15), and penicillin V (50,000 units/kg/day from day 18 until today). *Pneumocystis jiroveci *prophylaxis was performed with cotrimoxazol (5 mg/kg/day; 3 days per week from 1 until 6 months after BMT). Immunoglobulins were given at a dose of 250 mg/kg/day on days 2, 8, and 19. The patient received a total of 7 platelet concentrates, the last on day 13. The girl was discharged on day 22 in good clinical condition with a WBC of 1.2 × 10^9^/L, platelets of 32 × 10^9^/L, and hemoglobin of 6 mmol/L.

### Outcome

Blood chimerism analysis showed 100% donor cells on day 46. The percentage of donor cells declined to 91% on day 60 but resurged to 100% following withdrawal of cyclosporin A. Complete chimerism was established within 4 months after BMT, remaining stable since that time. As shown in figure 4, sequential flow cytometric studies of mitogen response *vs*. radiosensitivity confirmed the replacement of radiosensitive patient cells by radioresistant donor cells. Currently at age 16, her immune system functions well, with a good immune response towards all common vaccinations. During the course of two years after BMT the patient gained considerable weight. She had always been below the 20^th ^percentile, whereas now at age 16 she is overweight with 44 kg at 146 cm. Head circumference is 44 cm. Her height is still below the 3^rd ^percentile, but her general physical condition is excellent. She is fully active and can lead a normal life with no restrictions (100% Lansky-Play-performance). Due to her mild to moderate mental retardation, she currently attends grade 9 of a special school for mentally handicapped children, with good success. There are no skin or genital abnormalities, but puberty is markedly delayed. There is little pubic hair and breast development, corresponding to Tanner stages P3 and B3, and the patient has not yet started menstruating. The hormonal profile is suggestive of hypergonadotrophic hypogonadism (FSH is 55.5 IU/L, LH 24.2 IU/L, estradiol 73 pmol/L).

## Discussion

The clinical history of our patient illustrates the difficulty of establishing the correct diagnosis in rare growth failure and dysmorphy syndromes. The key observations that finally lead to the correct diagnosis were increased cellular sensitivity towards the radiomimetic bleomcycin (in terms of chromosome breakage), and increased cellular sensititvity to ionizing radiation (in terms of G2 phase cell cycle blockage). After exclusion of the Nijmegen breakage syndrome by mutation analysis and exclusion of AT on clinical grounds (supported by normal AFP levels), sequencing of the *LIG4 *gene established the diagnosis of DNA ligase IV deficiency syndrome. Due to the extensive phenotypic overlap it is conceivable that patients with features of the Dubowitz syndrome who in addition suffer from aplastic anemia [14,15] might eventually turn out to be affected by the DNA ligase IV deficiency syndrome.

As offspring of a non-consanguinous couple, our patient was diagnosed as compound heterozygous for an amino acid change (glycine replaced by glutamate) on the maternal allele and a stop codon mutation on the paternal allele [1]. Analysis of the impact of the R814X ligase IV mutation *in vivo *(hamster cell experiments) revealed an impaired interaction between ligase IV and XRCC4 (X-ray repair complementing defective repair in Chinese hamster cells 4 protein), and an impaired DNA ligase IV adenylate complex formation with reduced ligation activities [19]. When the G469E ligase IV mutation was analyzed in several experiments, a normal interaction with XRCC4 was observed, but adenylation and ligation activity were severely reduced, with very little residual ligation activity [19]. These results suggest that, at least in hamster cells, both mutations are pathogenic. Since LIG4 null-mutant mice are embryonic lethal [20] and biallelic null mutations have not been described to date in other LIG4-deficient patients [21,22], viability of the DNA ligase IV deficiency syndrome appears to require at least one allele with a hypomorphic mutation. A preserved pattern of TCR-alpha and beta junctions in the T cells and only mild to moderate impairment of class switch and V(D)J recombination suggests the presence of residual LIG4 activity in DNA ligase IV deficient patients [21,22]. The relatively mild degree of immunodeficiency observed in our patient agrees with the notion of residual LIG4 function as prerequisite for viability. However, at least some LIG4 deficient patients exhibit a severely compromised immune system, including complete absence of B lymphocytes and severely reduced numbers of T cells (SCID phenotype; [21,22]). This suggests that DNA ligase IV, beyond its well established role in V(D)J recombination, may have additional functions in the lymphocytic cell lineage [22].

Together with the XRCC4 protein, DNA ligase IV functions in non-homologous end joining (NHEJ), the major repair process of double strand breaks in mammalian cells [23]. DNA repair *via *NHEJ takes place during G0, G1, and early S phase of the cell cycle and requires Ku (a Ku70/Ku80 heterodimer), DNA-dependent protein kinase catalytic subunit (DNA-PKcs), and the XRCC4/LIG4 complex proteins [24-26]. It is assumed that Ku or Rag binds to the DSB first, then recruits DNA-PKcs and Artemis, a nuclease that trims DNA ends allowing the LIG4/XRCC4 complex to perform religation [24-26]. In addition, NHEJ is essential for the generation of T cell receptor and immunoglobulin diversity. Impairment of class switch and of V(D)J recombination therefore explain the immunodeficiency and proneness to multiple myeloma and lymphoma observed in LIG4-deficient patients [17,26]. It does not, however, explain the propensity to bone marrow failure. In analogy to the bone marrow failure syndromes Fanconi anemia and Dyskeratosis congenita which are characterized by instability of chromosomes or telomeres [27,28], genetic instability due to LIG4 deficiency might explain increased radiosensitivity and bone marrow failure in LIG4-deficient patients.

It has previously been shown in mice that ligase IV is essential for neuronal cell development [29]: Disruption of NHEJ due to ligase IV deficiency leads to impaired prenatal differentiation of neuronal cells. This may explain the mild to moderate mental retardation observed in our patient.

Like in Fanconi anemia and a single previously reported patient with the Nijmegen breakage syndrome, our patient illustrates that HSCT can be an effective treatment of LIG4-deficiency. Since our patient had a matched sibling donor available, a six-year history of progressive bone marrow failure requiring increasing rates of blood product substitution, and since she showed signs of developing myelodysplastic syndrome (MDS), HSCT was the most promising therapeutic option. It should be pointed out, however, that HSCT in our LIG4-patient was prompted by bone marrow hypo-and dysplasia rather than by a SCID phenotype as in other patients with LIG4 syndrome [22,16].

To our knowledge, this is the first detailed description of a modified conditioning regimen for BMT and of the clinical course after transplantation in a Ligase IV deficiency patient. In other publications [1,4] BMT is briefly mentioned, but neither the procedure nor the outcome have been described in detail in any of the previous patients. Due to our patient's cellular radiosensitivity, she most likely would not have tolerated a conditioning regimen using total body irradiation and high dose cyclophosphamide. In addition, such aggressive conditioning would have increased her risk of secondary malignancies [30]. Our protocol therefore used a mild conditioning regimen without any irradiation, a reduced dose of cyclophosphamide, and no methotrexate for GvHD prophylaxis. This reduced toxicity lead to rapid engraftment without severe side effects, complete and stable chimerism at 4 months, and good clinical condition at four years after transplant.

## Conclusion

HSCT using mild conditioning without irradiation qualifies as treatment of choice in LIG4-deficient patients who have a matched sibling donor. In addition, the development of bone marrow failure in our patient suggests that at least some forms of Ligase IV deficiency belong to the inherited bone marrow failure syndromes.

## Abbreviations

BrdU = bromodesoxyuridine; GvHD = graft versus host disease; FSH = follicle stimulation hormone; LH = luteinizing hormone; AFP = alpha-fetoprotein; TCR = T-cell receptor; SCID = severe combined immunodeficiency disease; MDS = myelodysplastic syndrome

## Competing interests

The author(s) declare that they have no competing interests.

## Authors' contributions

BG was the attending physician performing BMT. FZ and JS were responsible for patient care and follow up. The Berlin group did cytogenetic and Bleomycin testing as well as the mutation analysis. The Wurzburg group carried out the flow cytometric testing. AB and DS were responsible for writing the manuscript.
